# Impact of game mode in multi-user serious games for upper limb rehabilitation: a within-person randomized trial on engagement and social involvement

**DOI:** 10.1186/s12984-019-0578-9

**Published:** 2019-08-30

**Authors:** Fábio Pereira, Sergi Bermúdez i Badia, Rúben Ornelas, Mónica S. Cameirão

**Affiliations:** 1 Faculdade de Ciências Exatas e da Engenharia, Universidade da Madeira, Campus Universitário da Penteada, 9020-105 Funchal, Portugal; 2 ITI-LARSyS and Madeira ITI, Pólo Científico e Tecnológico da Madeira, Caminho da Penteada, 9020-105 Funchal, Portugal

**Keywords:** Serious games, Game mode, Engagement, Rehabilitation, Social involvement, Elderly

## Abstract

**Background:**

Serious games have been increasingly used for motor rehabilitation. However, it is not well known how different game features can be used to impact specific skills properly. Here, we study how the mode (competitive, co-active, collaborative) in which a multi-user game is presented impacts engagement and social involvement.

**Methods:**

We collected data from 20 pairs of community-dwelling older adults (71.5 ± 8.7 years) in a study following a within-persons design. The participants performed a two-player upper limb rehabilitation game with three conditions (Competitive, Co-active, and Collaborative modes). Engagement and social involvement were assessed through the Core Module and Social Presence Module, respectively, from the Game Experience Questionnaire. To infer the impact of personality and cognitive function, users answered the International Personality Item Pool (short version) and the Montreal Cognitive Assessment, respectively.

**Results:**

Results show that the Collaborative game mode promotes more social involvement when compared to Competitive and Co-active modes. This result is mostly explained by those participants with higher cognitive skills, and those that are more extrovert. Extrovert participants feel more empathy and are behaviorally more involved when playing the Collaborative mode. Also, the Collaborative mode is shown to be appropriate to promote interaction with participants that previously had a distant relationship, while the Competitive mode seems to be more beneficial to promote empathy between players with a closer relationship.

**Conclusions:**

The Collaborative game mode elicited significantly higher social involvement in terms of Empathy, Positive Affect, and Behavioral Involvement. Hence, this game mode seems to be the most adequate choice to be used in multiplayer rehabilitation settings, where social interaction is intended.

## Background

Serious games have been widely studied concerning their impact on improving physical and social skills with elderly [1], mostly because of their potential to increase motivation levels compared to conventional therapies [2, 3]. Moreover, the therapeutic potential of games for the elderly is well documented, with results showing a positive impact on their health and well-being [1]. However, while one of the most critical elements of successful aging is to conserve social relationships [4], research on how to appropriately address social experiences with serious games is still scarce [1]. Also, social interaction through multiplayer games has been underlined as an essential aspect of motor rehabilitation because it supports enhanced enjoyment during interaction and an increased sense of self-efficacy [5]. In fact, stroke survivors with low levels of social support have a greater risk of developing depression [6]. A longitudinal study examined the impact of social support in 5643 participants that had experienced a heart attack or stroke, with results indicating that the risk of developing depression is very contingent to the level of social support [6]. Moreover, social support is a modifiable factor that can mitigate the impact of illness on depression, and higher social support could improve the outcomes [6]. In another study, Janssen et al.*,* investigated how social activity of stroke patients undergoing rehabilitation changes over time [7]. After analyzing data from a sample of 14 participants, the authors concluded that the levels of social activity were low even after improvements in the levels of independence and mood. These data highlight the need to explore alternative ways of social stimulation within rehabilitation environments [7].

One way to foster social interaction is through multiplayer modalities, which typically promote more socialization than their single-player counterparts [8]. For rehabilitation purposes, the design and specific characteristics of these games should be carefully analyzed, to identify the features that influence motivation and engagement levels, which in turn could have an impact on recovery. One feature that potentially can have an impact is the playing mode. I.e., inter-player relationships can be of one of the following four kinds of interaction: competitive, co-active, cooperative or collaborative [9, 10]. However, the literature typically addresses competitive, collaborative and cooperative modes only [11]. In addition, most of the studies that addressed game modes focused on competitive vs. cooperative, or competitive vs. collaborative, not establishing a difference between cooperative and collaborative modes [8, 12–16]. The main difference between cooperation and collaboration is that cooperation requires players to work together to complete a task but having different roles, while collaboration implies players to have the same role and still needing to work together to complete the task [9, 17]. Moreover, there is no consistency in nomenclature. Some modes of training identified in previous studies should be named as co-active instead of cooperative, as they imply that players work together to reach the same goal with the same task, but they do not depend on each other to finish it [9, 10]. Considering these different definitions, research on collaborative games is lacking. To our best knowledge, only a minority of studies differentiated the three above mentioned game modes [18], and according to a systematic review on multi-player games, there is no research comparing them [11].

Multiplayer rehabilitation games show good potential for producing greater enjoyment and more intense exercise in comparison to single-player modalities [8]. A study with 12 pairs of unimpaired participants concluded that participants prefer to cooperate than to exercise alone, feeling less pressure in this mode [19]. Another study that linked patients with their spouses in rehabilitation through haptic interaction found that multiplayer modes were more motivating when compared to single-player modes [20]. Nevertheless, the right game mode, when comparing competitive vs. collaborative modes, for a specific person depends on skill and personality, alongside with having an appropriate co-player [8]. A study with 158 healthy adults found that a co-active mode caused higher levels of motivation and effort in comparison with a competitive mode, but motor performance was similar in both [21]. An interesting result of this study was that the level of relationship (friends vs. strangers) also influenced players’ motivation, goal commitment, and performance. Those who played with friends showed greater goal commitment than those who played with strangers [21]. In contrast, several studies indicate that competitive modes motivate players more, resulting in more intense performance, and are associated with more movement repetitions [12, 15, 22, 23].

Here, we aim to understand the impact on engagement and social involvement of different multiplayer settings, specifically aiming to distinguish collaboration and cooperation. For this purpose, we deployed a game in three different modes (Competitive, Co-active and Collaborative) and tested these in a sample of healthy older adults (> 55 years). We decided for a sample of older adults without motor deficits to avoid potential confounds brought by stroke, which is known to impact cognitive, motor, emotional or social domains. We believe that a sample where these domains remain relatively intact represents a good baseline to assess the impact of these game modes on engagement and social involvement. Next, with a stroke population it will be possible to better understand how these results are modulated by the deficits brought by stroke. Our first hypothesis is that engagement will be significantly higher in the Competitive mode when compared to Co-active and Collaborative modes. Our second hypothesis is that social involvement will be significantly higher in the Collaborative game mode compared to Competitive and Co-active modes. Additionally, we want to understand how the results are modulated by the cognitive profile, personality, and previous relationship between co-players.

## Methods

### Pilot testing

**Fig. 1:**
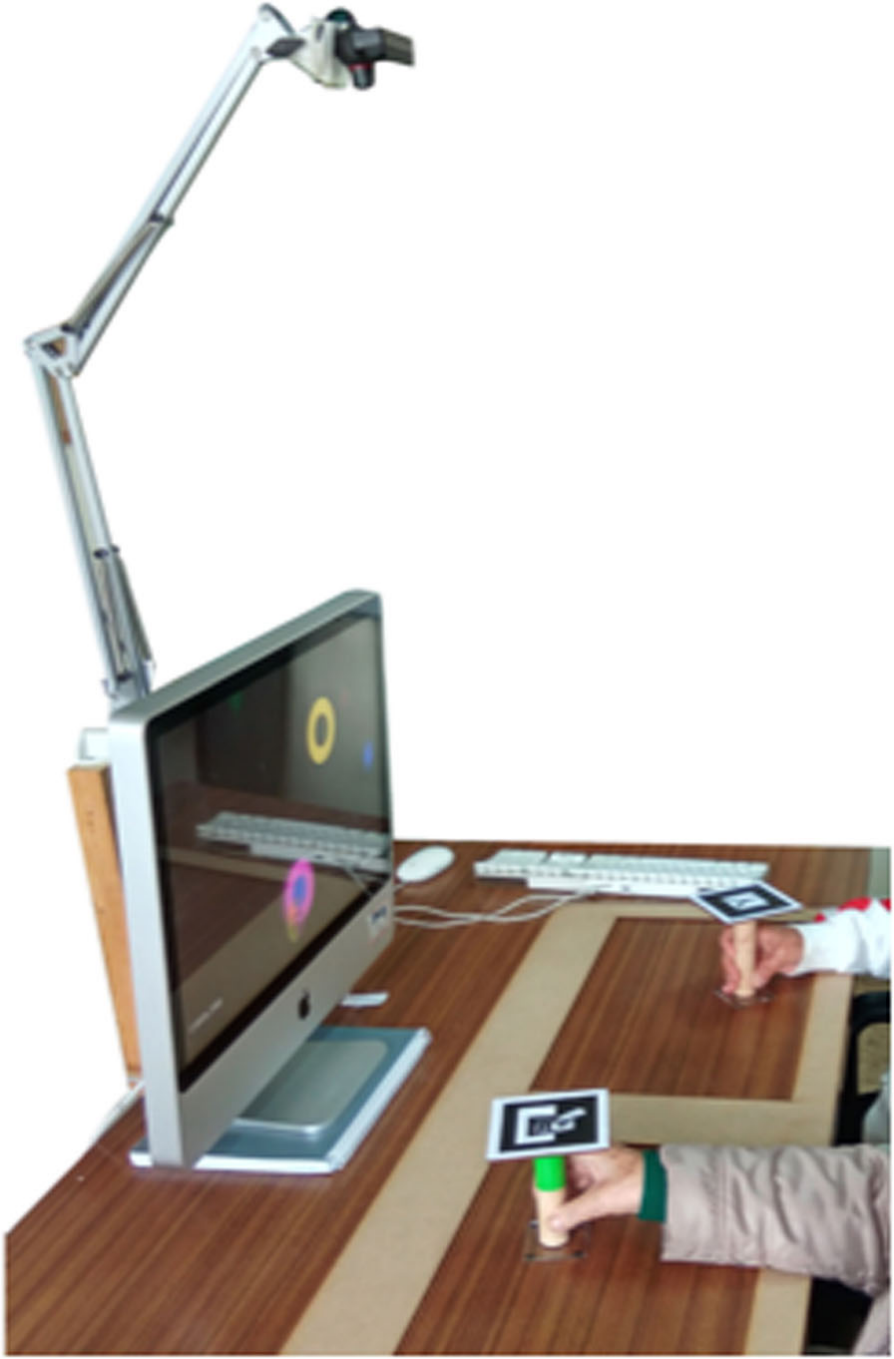
Setup with a camera, camera support, computer, handles with tracking pattern, and wood card for delimiting the working space

Before the final experiment, three pilot tests were conducted to address game interaction and mechanics, and understandability issues of the setup and assessment questionnaires. Four participants (4 female), with a mean age of 79 years (range 60–86 years), and 3–7 years of schooling participated in these pilots. As a result, changes were made in the interaction form, starting with a joystick and ending with a handle (interface) that worked similarly to a mouse (Fig. 1). Also, the Collaborative mode was changed to simplify mechanics. Regarding the assessment questionnaires, some points were reformulated to become more understandable.

### Experimental setup

The setup consisted of a PC (OS: Windows 7, CPU: Intel core 2 duo E8235 at 2.80GHzz, RAM: 4Gb, Graphics: ATI Mobility Radeon HD 2600 XT) with a 24″ screen, a PlayStation Eye camera (Sony Computer Entertainment Inc., Tokyo, Japan) and two customized handles with a tracking pattern (Fig. 1). For tracking, we used Analysis and Tracking System (AnTS) [24], which uses computer vision to detect specific patterns on the camera’s field of view. For that, we printed two patterns and attached them to handles and used them as controllers, as the pattern positions are mapped on the screen space. Users were seated side-by-side and facing the screen. To interact with the game, users had to grasp the handle and move it over the surface of the table. A 3 mm wood card with two cut down rectangles (50 cm by 30 cm) was used to delimit the space where the camera was tracking the handles. These rectangles made the calibration process easier and reliable, guaranteeing an equal calibration for all the participants. The calibration was done using the Reh@Panel Unity3D client (https://neurorehabilitation.m-iti.org/tools/en/rehapanel-unity-client) and consisted of mapping the X and Y limits where the handle could move to the whole screen area.

### Task

**Fig. 2:**
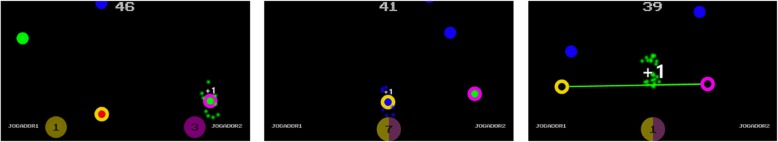
Competitive, Co-active and Collaborative game modes, from left to right. Each player controls a ring (yellow or pink) to catch balls of different colors (the center of the ring shows the color of the last ball caught). The screen displays on the top the remaining time and on the bottom the name of the players and their respective score (matching the color attributed to each player). In the Competitive mode, players compete for the balls, in the Co-active mode players can catch any ball for a combined score, and in the Collaborative mode, when one of the participants catches a ball of a given color (eg. green), then the other player can only catch a ball of the same color (green). For this mode, when scoring, a line between the two rings is formed to give feedback that players succeeded

The task was a two-player game with the primary objective of catching balls that are falling from the top of the screen. For that purpose, each user controlled a virtual ring on the screen by moving the handle on the table. There were three different versions of the task, which corresponded to three game modes: Competitive, Co-active, and Collaborative (Fig. 2). Differences between the game modes relied on the objectives, but the task mechanics were the same. In the Competitive mode, participants had to catch the maximum number of balls. The participant who scored more points (each ball resulted in 1 point) won the round. In the Co-active game mode, participants had to play as a team and catch balls for a combined score. In the Collaborative game mode, they also played as a team; nevertheless, for participants to score in this mode, both had to catch a ball of the same color. If one of the participants caught a ball of a specific color (eg. green), then the other player could only catch a ball of the same color (green). As a consequence of scoring, a line between the two rings was formed to give feedback that players succeeded. There was no in-game adaptation according to the participants performance. The game settings were chosen to make sure that the task was doable by all our participants.

### Experimental procedure

The study followed a within-person design with three independent variables (Competitive, Co-active, and Collaborative) to assess the impact on engagement and social involvement. The order of the conditions was previously randomized using *random.org*, and sample allocation was according to the availability of participants. Data collection was conducted in two sessions of approximately one hour each and ran in different days or morning-and-afternoon sessions, according to the availability of the participants. In average, the time between the two sessions was 79 h (3 days). The sessions were conducted by two researchers trained on the system and the assessment questionnaires. In the first session, participants were organized in pairs, introduced to the study, got familiarized with the game, signed the informed consent, and performed the first condition. In the second session, participants performed the remaining two conditions. Each condition consisted of 8 consecutive rounds of 1-min duration with a 5–15 s interval between rounds to allow participants to interact spontaneously. Between each round, the score was reset. At the end of each condition, participants answered the GEQ – Core Module and GEQ – Social Presence Module [25]. The total time of the experiment was about 90–150 min, depending on the participant, because of the variable times to apply the questionnaires.

After a brief explanation of the system, participants underwent two training phases. The first one was to learn how to use the interface and how to control the virtual ring (end effector). This training phase did not have a time limit, ending when participants felt comfortable controlling the virtual ring (as assessed by the researcher). In a second training phase, participants tried each game mode mechanics until they felt they had understood it, also aiming to reduce the novelty bias. Here, we also decided not to restrict the time, as each participant needed different times and feedback to understand and get used to what was expected from him/her.

### Sample and recruitment

The sample was recruited at a community center that provides social support to the population. It was a convenience sample, and the single inclusion criterium was to be more than 55 years old. The exclusion criteria were the following: 1) Motor limitations in the dominant upper limb; 2) no literacy; and 3) not able to understand the game according to the therapist’s assessment [26]. Fifty-five potential participants were approached. Seven refused to participate, and eight were excluded due to exclusion criteria. Forty participants (20 pairs) took part in the study. Out of these, the data of 1 participant were not considered because of lack of compliance during the assessment questionnaires, leaving a final sample of 39 participants for analysis. Twenty-four were females and 15 were males, with a mean age of 71.5 ± 8.7 years (range: 56–91 years) and 6.2 ± 4.2 years of schooling (range: 2–16) (Table 1).

**Table 1:** Participants’ profile

Participant	Gender (M/F)	Age	Schooling (Years)	MoCA (0–30)
1	M	56	7	28
2	F	62	12	25
3	F	81	7	16
4	F	71	7	14
5	F	63	7	16
6	F	72	4	12
7	M	70	7	16
8	F	76	16	25
9	M	64	16	28
10	M	75	7	23
11	M	66	7	17
12	M	77	7	16
13	M	73	6	20
14	F	83	15	23
15	F	61	7	25
16	F	69	16	21
17	M	58	7	24
18	F	71	7	17
19	F	63	16	28
20	F	70	16	24
21	F	80	7	16
22	M	65	7	30
23	F	62	7	26
24	M	91	16	19
25	F	73	6	18
26	M	85	7	24
27	F	62	16	26
28	M	68	7	23
29	F	63	16	26
30	F	75	6	15
31	M	72	7	14
32	F	72	16	24
33	M	77	12	22
34	M	86	16	26
35	F	63	7	21
36	F	77	7	27
37	F	66	7	22
38	F	87	12	17
39	F	85	7	15

To get a profile of the participants, we used: 1) a brief questionnaire for demographic information; 2) a Likert scale (from 1 = distant relationship to 10 = very close relationship) for a self-reported characterization of the previous relationship between pairs; 3) the Montreal Cognitive Assessment (MoCA) for cognitive screening [26]; and 4) the Mini-IPIP validated for Portuguese population [27] to characterize the participants with respect to their personality. Although Mini-IPIP measures the Big Five factors of personality (Openness/Intellect or Imagination, Conscientiousness, Extraversion, Agreeableness, and neuroticism, only the extraversion factor was analyzed since to our understanding it is the one that relates clearly to engagement and social involvement. The mean value for MoCA was 21.3 ± 4.9 (range: 12–30). Additionally, regarding previous experience with digital games, 38.5% of the sample had previous experience with video games. From these, 44% played once or twice per year, 25% every week, and 31% daily. The computer was the most used interface (55%), followed by smartphones, tablets, and video game consoles.

### Outcome measures

The Game Experience Questionnaire (GEQ) – Core Module [25] and the GEQ – Social Presence Module [28] were chosen to measure engagement and social involvement, respectively. The Core Module measures the players’ thoughts and feelings through 7 components (Competence, Sensory and Imaginative Immersion, Flow, Tension/Annoyance, Challenge, Negative affect, and Positive affect) in a total of 33 items [25]. The Social Presence Module has three components (Psychological Involvement – Empathy, Psychological Involvement – Negative Feelings and Behavioral Involvement), and a total of 17 items. In both questionnaires, the items are rated from '0' (Not at all) to '4' (Extremely). These questionnaires are typically filled-in by the user, but because of the characteristics of the sample, the answers’ scale was provided on an A4 sheet, always visible to the participants, and the questions were made verbally. The scale was translated from English to Portuguese by two experts in English-Portuguese translation.

### Data analysis

Because of the ordinal nature of the measures, non-parametric statistical tests were used for data analysis. Hence, the median was used as a measure of central tendency and the interquartile range (IQR) for dispersion. To test for differences across conditions, we used Friedman’s test for each of the components of both modules from the GEQ. We tested for significant differences across game modes in the three groups. For pairwise comparisons, the Wilcoxon signed-rank test was used with a Bonferroni correction to account for the number of comparisons. To analyze how cognitive skills and personality impacts engagement and social involvement in the three game modes, data were split into subgroups. For cognitive level, we divided the sample into two subgroups using the mean of MoCA scores (21.26 ± 4.86): ‘Higher MoCAs’ (score above 21) (*n* = 21) and ‘Lower MoCAs’ (score lower or equal to 21) (*n* = 18). For personality, we computed the mean of extraversion component of Mini-IPIP (11.54 ± 3.64) and then summed and subtracted half of the standard deviation as suggested by the official website for the International Personality Item Pool [29]. This way, we got a range (9.72–13.36) where average people fall in (*n* = 16). Below that range, people are considered more introvert (*n* = 11) and above the range, more extrovert (*n* = 12) than the average. Finally, to analyze the effect of co-player relationship, we divided the sample into two subgroups, according to the score attributed by each participant to their level of relationship (1 (no relationship) -10 (close relationship)) with the correspondent pair. The first subgroup (*n* = 22) was composed of participants that considered having a distant relationship, attributing a score lower or equal to 5. The second subgroup (*n* = 17), was composed of participants that attributed a score higher than 5, indicating a closer relationship. For between-group comparisons, we used the Mann-Whitney U Test or the Kruskal-Wallis Test, for pairwise or more than two conditions, respectively. Data were analyzed using IBM Statistics for Mac, Version 25.0 (Armonk, NY: IBM Corp).

## Results

### Social involvement

**Fig. 3:**
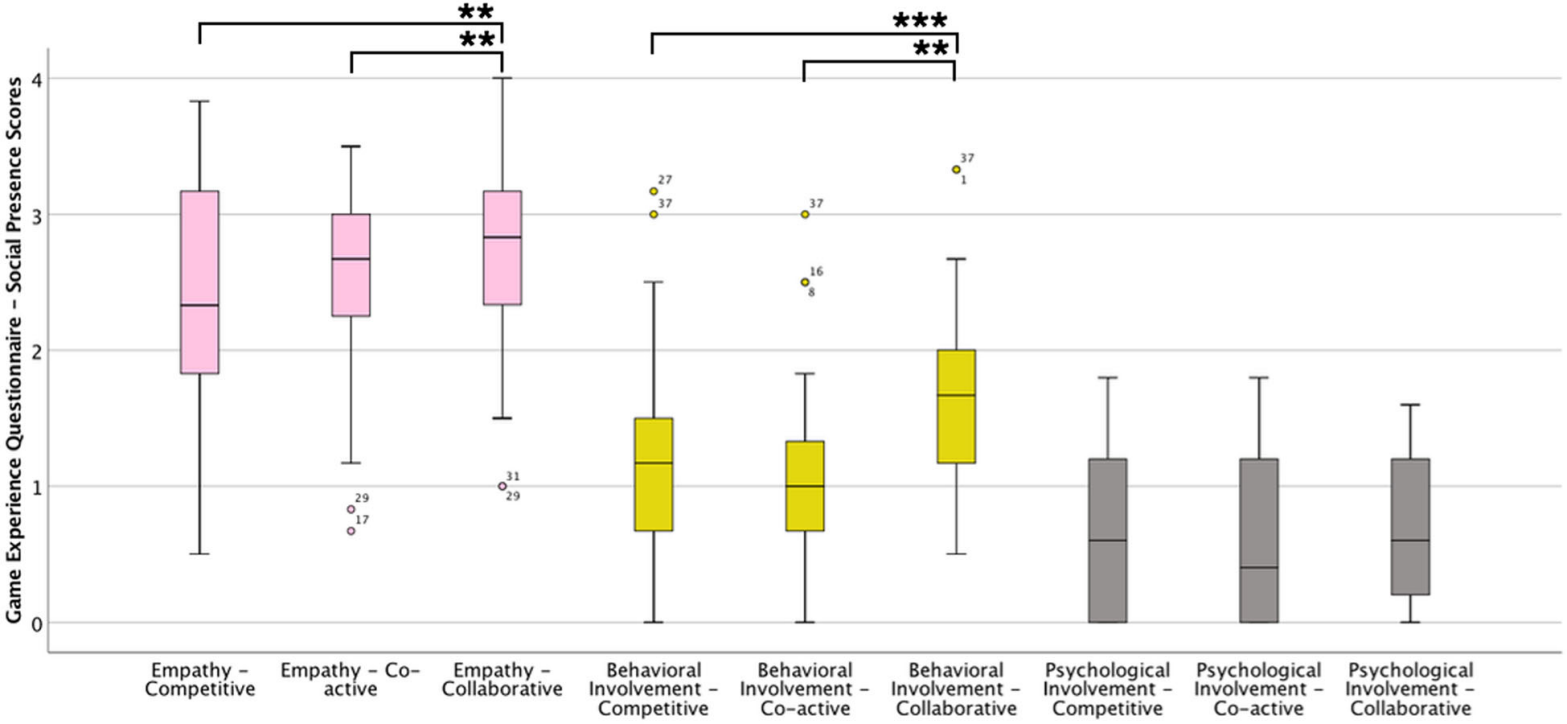
Boxplots of the GEQ – Social Presence Module components per game mode. ** *p* < 0.01, and ***p < 0.001

Regarding the GEQ Social Presence Module, Empathy was relatively high on all conditions (Mdn = [2.33–2.83]), but with a lower median and higher dispersion in the Competitive mode (Fig. 3, Table 2). The difference across conditions for this component was significant (Fr(2) = 6.587, *p* = 0.037), with the Collaborative mode having a significantly higher rating when compared to Co-active (Z = -3.723, *p* = 0.006) and Competitive (Z = -2.885, *p* = 0.004) modes (Table 3). For Behavioral Involvement, ratings tended to be low (Mdn = [1.00–1.67]), and there was a significant effect across conditions (Fr(2) =18.440, *p* < 0.001). Also, for this component, the Collaborative mode showed a significantly higher rating when compared to Co-active (Z = -4.068, *p* = 0.000) and Competitive (Z = -3.273, *p* = 0.001) modes. Finally, ratings were low for Negative Feelings (Mdn = [0.40–0.60]), with no significant differences across conditions (Table 3).

**Table 2:** Medians (IQR) in the components of the Game Experience Questionnaire – Social Presence Module per condition, and Friedman’s statistics across conditions

Component	Competitive	Co-active	Collaborative	Friedman’s statistic	
				(Chi-Square)	*p* value
Empathy	2.33 (1.34)	2.67 (0.83)	2.83 (1.00)	6.587	**0.037**
Behavioral Involvement	1.17 (0.83)	1.00 (0.66)	1.67 (0.83)	18.440	**< 0.001**
Negative Feelings	0.60 (1.20)	0.40 (1.20)	0.60 (1.00)	0.271	0.873

Bold values represent that significant differences between conditions were found

**Table 3:** Pairwise comparisons for Empathy and Behavioral Involvement components

Component		Competitive Vs Co-active	Competitive Vs Collaborative	Collaborative Vs Co-active
Empathy	Z	−0.941	−2.885	−2.723
	*p* value	0.347	**0.004**	**0.006**
Behavioral Involvement	Z	−1.030	−3.273	−4.068
	*p* value	0.303	**0.001**	**< 0.001**

Bold values represent that significant differences between conditions were found

### Engagement

Concerning the components of the GEQ Core Module, Flow was high for all the conditions (Mdn = 2.80–3.00) and so was Positive Affect (Mdn = 3.00) (Table 4). However, the task was not considered challenging enough (Challenge, Mdn = 0.60–0.80). Although the sense of Competence was in general not very high (Mdn = 2.20–2.40), feelings of Tension/Annoyance were very low (Mdn = 0.00). No significant differences were found when comparing conditions in these domains (Table 4).

**Table 4:** Medians (IQR) in the components of the GEQ – Core Module per condition, and Friedman’s statistics across conditions

Component	Competitive	Co-active	Collaborative	Friedman’s statistic	
				(Chi-Square)	*p* value
Competence	2.40 (1.40)	2.60 (1.20)	2.20 (1.20)	0.557	0.757
Sensory and Imaginative Immersion	2.33 (1.17)	2.50 (1.00)	2.33 (1.00)	0.529	0.768
Flow	2.80 (1.20)	2.80 (1.20)	3.00 (1.00)	3.418	0.181
Tension/Annoyance	0.00 (0.00)	0.00 (0.00)	0.00 (0.00)	1.182	0.554
Challenge	0.80 (0.60)	0.60 (1.00)	0.80 (1.00)	4.211	0.122
Negative Affect	0.00 (0.19)	0.00 (0.00)	0.00 (0.00)	5.688	0.058
Positive Affect	3.00 (1.20)	3.00 (0.80)	3.00 (0.80)	0.803	0.669

### Effect of cognitive profile

For the Higher MoCAs subgroup, significant differences across conditions were found for the Behavioral Involvement component (Fr(2) = 13.468, *p* = 0.001) of the Social Presence Module (Table 5). Further pairwise comparisons showed that the Collaborative mode had significantly higher ratings (Mdn = 1.83 (0.91)) than the Competitive mode (Mdn = 1.33 (1.00)) (Z = -2.567, *p* = 0.010) and the *Co-active* mode (Mdn = 1.00 (0.58)) (Z = -3.515, *p* < 0.001). There were no significant differences across conditions for the different components of the Core Module (Table 5).

**Table 5:** Medians (Mdn) and Interquartile Range (IQR) for higher and lower MoCAs for each condition (game mode), between-groups comparison (Mann-Whitney U Test), and Friedman’s statistics for higher and lower MoCAs according to each component of the Game Experience Questionnaire’s Core Module and Social Presence Module

		Competitive			Co-active			Collaborative			Between conditions comparison	
	Component	Higher MoCAs	Lower MoCAs	Between-groups comp.	Higher MoCAs	Lower MoCAs	Between-groups comp.	Higher MoCAs	Lower MoCAs	Between-groups comp.	Higher MoCAs	Lower MoCAs
		Median (IQR)	Median (IQR)	Mann-Whitney U, *p* Value	Median (IQR)	Median (IQR)	Mann-Whitney U, *p* Value	Median (IQR)	Median (IQR)	Mann-Whitney U, *p* Value	Chi-Square, *p* Value	Chi-Square, *p* Value
Core Module	Competence	2.40 (2.00)	2.40 (1.10)	162.5, 0.454	2.80 (1.10)	2.20 (0.95)	154.5, 0.329	**3.00 (1.40)**	**2.20 (0.75)**	**118.5, 0.046**	0.444, 0.801	1.971, 0.373
	Sensory and Imaginative Immersion	2.50 (1.00)	2.09 (1.21)	304.5, 0.117	2.50 (1.17)	2.33 (0.88)	322.0, 0.283	**2.67 (1.09)**	**2.09 (0.88)**	**286.5, 0.038**	2.111, 0.348	0.206, 0.902
	Flow	**3.20 (1.10)**	**2.40 (1.30)**	**116.5, 0.040**	3.00 (1.20)	2.80 (1.30)	147.5, 0.240	**3.20 (1.10)**	**2.50 (0.85)**	**100.0, 0.012**	3.909, 0.142	0.471, 0.790
	Tension/Annoyance	0.00 (0.00)	0.00 (0.00)	185.0, 0.830	0.00 (0.00)	0.00 (0.00)	167.5, 0.250	0.00 (0.00)	0.00 (0.00)	187.0, 0.883	0.800, 0.670	0.500, 0.779
	Challenge	0.80 (0.60)	0.70 (1.10)	162.5, 0.451	0.60 (1.00)	0.40 (1.25)	161.0, 0.426	1.00 (0.90)	0.60 (0.85)	154.5, 0.328	2.413, 0.299	3.033, 0.219
	Negative Affect	0.00 (0.13)	0.00 (0.06)	186.0, 0.909	0.00 (0.00)	0.00 (0.00)	186.5, 0.894	0.00 (0.00)	0.00 (0.00)	175.5, 0.512	4.769, 0.092	2.632, 0.268
	Positive Affect	3.40 (1.10)	3.00 (0.65)	136.0, 0.131	3.20 (0.70)	3.00 (0.60)	125.0, 0.068	3.40 (1.00)	3.00 (0.50)	122.0, 0.057	0.488, 0.799	0.375, 0.829
Social Presence Module	Empathy	2.50 (1.16)	2.17 (1.54)	150.5, 0.277	2.67 (0.50)	2.42 (1.00)	138.5, 0.151	2.83 (0.75)	2.92 (1.12)	167.0, 0.533	2.960, 0.228	5.559, 0.062
	Negative Feelings	0.60 (1.20)	0.60 (0.70)	181.0, 0.819	0.40 (1.20)	0.50 (1.05)	188.0, 0.977	0.40 (1.00)	0.80 (1.20)	180.0, 0.796	0.500, 0.779	0.030, 0.985
	Behavioral Involvement	**1.33 (1.00)**	1.09 (0.91)	159.5, 0.404	**1.00 (0.58)**	0.67 (1.34)	165.5, 0.505	**1.83 (0.91)**	1.42 (1.08)	127.0, 0.079	**13.468, 0.001**	5.548, 0.062

Bold values represent that significant differences between conditions were found

Concerning the Lower MoCAs subgroup, there were no significant differences across conditions for the different components of the Core and the Social Presence Modules (Table 5).

When comparing the two MoCA subgroups for each game mode, ratings were typically higher for the higher MoCAs subgroup (Table 5). There were between-group significant differences in the Collaborative mode for the sense of Competence (U = 118.500, *p =* 0.046), Sensory and Imaginative Immersion (U = 115.500, *p =* 0.038), and Flow (U = 100.00, *p =* 0.011). For the Competitive mode, the higher MoCAs subgroup had significantly higher ratings in Flow (U = 116.500, *p =* 0.040).

### Effect of personality

The ‘extrovert’ group showed significant differences across conditions in the Behavioral Involvement component of the GEQ – Social Presence Module (Fr(2) = 12.049, *p* = 0.002) (Table 6). Further pairwise comparisons showed that the Collaborative mode (Mdn = 1.67 (0.81)) promotes significant more Behavioral Involvement than the Competitive (Mdn = 0.83 (0.99)) (Z = -2.496, *p* = 0.013) and Co-active (Mdn = 1.08 (0.83)) (Z = -2.938, *p* = 0.003) modes. Also, the Collaborative mode (Mdn = 3.09 (0.612)) promotes significantly more empathy than the Competitive (Mdn = 2.17 (0.89)) (Z = -2.502, *p* = 0.012) and Co-active (Mdn = 2.67 (0.61)) (Z = -2.858, *p* = 0.004) modes.

**Table 6:** Medians (Mdn) and Interquartile Range (IQR) for Introvert, Average and Extrovert personalities for each condition (game mode), between conditions comparison (Friedman’s) for the three personalities, according to each component of the GEQ Core Module and Social Presence Module

		Competitive			Co-active			Collaborative			Between conditions comparison		
	Component	Introvert	Average	Extrovert	Introvert	Average	Extrovert	Introvert	Average	Extrovert	Introvert	Average	Extrovert
		Mdn (IQR)	Mdn (IQR)	Mdn (IQR	Mdn (IQR)	Mdn (IQR)	Mdn (IQR)	Mdn (IQR)	Mdn (IQR)	Mdn (IQR)	Chi-Square, *p* Value	Chi-Square, *p* Value	Chi-Square, *p* Value
Core Module	Competence	2.80 (0.89)	2.20 (0.84)	2.50 (1.00)	3.00 (0.79)	2.20 (0.68)	2.40 (0.97)	3.00 (0.73)	2.20 (0.68)	2.40 (0.73)	0.052, 0.974	1.368, 0.504	1.911, 0.385
	Sensory and Imaginative Immersion	2.67 (0.85)	2.17 (0.85)	2.25 (0.77)	2.67 (0.71)	2.17 (0.65)	2.34 (0.72)	2.50 (0.75)	2.17 (0.60)	2.42 (0.71)	0.195, 0.907	1.750, 0.417	1.302, 0.521
	Flow	2.60 (1.03)	2.80 (0.59)	3.20 (0.70)	2.80 (0.89)	2.80 (0.76)	3.30 (0.91)	3.20 (0.71)	2.60 (0.70)	3.00 (0.71)	**6.545, 0.038**	1.793, 0.408	0.884, 0.643
	Tension/Annoyance	0.00 (0.00)	0.00 (0.42)	0.00 (0.95)	0.00 (0.00)	0.00 (0.26)	0.00 (0.30)	0.00 (0.00)	0.00 (0.42)	0.00 (0.95)	-, -	2.000, 0.368	1.400, 0.497
	Challenge	0.60 (0.66)	1.00 (0.57)	0.90 (0.54)	0.20 (0.53)	0.70 (0.70)	0.50 (0.65)	0.40 (0.59)	0.80 (0.53)	0.90 (0.76)	1.400, 0.497	1.077, 0.584	2.780, 0.249
	Negative Affect	0.00 (0.15)	0.00 (0.50)	0.00 (0.25)	0.00 (0.00)	0.00 (0.20)	0.00 (0.29)	0.00 (0.75)	0.00 (0.44)	0.00 (0.14)	1.000, 0.607	3.500, 0.174	3.200, 0.202
	Positive Affect	3.40 (0.46)	3.00 (0.58)	3.00 (0.66)	3.00 (0.46)	3.00 (0.56)	3.30 (0.69)	3.20 (0.59)	2.80 (0.54)	3.00 (0.71)	3.353, 0.187	0.462, 0.794	0.222, 0.895
Social Presence Module	Empathy	2.67 (0.41)	2.08 (0.97)	**2.17 (0.89)**	2.67 (0.55)	2.59 (0.83)	**2.67 (0.61)**	3.00 (0.62)	2.83 (0.84)	**3.09 (0.62)**	0.474, 0.789	3.581, 0.167	**7.953, 0.019**
	Negative Feelings	0.80 (0.54)	0.50 (0.53)	0.40 (0.59)	0.60 (0.46)	0.30 (0.60)	1.10 (0.61)	0.40 (0.53)	0.40 (0.51)	1.10 (0.48)	1.471, 0.479	0.154, 0.926	0.437, 0.804
	Behavioral Involvement	1.50 (0.75)	1.17 (0.62)	**0.83 (0.99)**	1.00 (0.61)	1.00 (0.66)	**1.08 (0.83)**	1.50 (0.69)	1.50 (0.61)	**1.67 (0.81)**	2.513, 0.285	**7.311, 0.026**	**12.049, 0.002**

Bold values represent that significant differences between conditions were found

The ‘introvert’ group showed significant differences across conditions in the Flow component of the GEQ – Core Module (Fr(2) = 6.545, *p* = 0.038). Further pairwise comparisons showed no significant differences after Bonferroni correction. On what concerns the ‘average’ group, significant differences across conditions were found in the Behavioral Involvement component (Fr(2) = 7.311, *p* = 0.026), however, again after the Bonferroni corrections, pairwise statistics were not significant.

When comparing the three types of personalities for each game mode, no significant differences were found.

### Effect of previous relationship

Regarding the effect of previous relationship between players, significant differences across game modes were found on the Behavioral Involvement component for distant relationships (Fr(2) = 13.835, *p* = 0.001) and close relationships (Fr(2) = 6.533, *p* = 0.038) (Table 7). For distant relationships, pairwise comparisons showed that the Collaborative mode (Mdn = 1.75 (1.42)) promotes significant more Behavioral Involvement than the Competitive (Mdn = 0.92 (1.05)) (Z = -3.265, *p* = 0.001), and Co-active (Mdn = 1.00 (0.66)) (*Z* = -3.396, *p* = 0.001) modes. For the subgroup with a closer relationship, pairwise statistics were not significant after Bonferroni correction.

**Table 7:** Medians (Mdn) and Interquartile Range (IQR) for Distant and Close Relationships for each condition (game mode), between-groups comparison (Mann-Whithney U Test), and between conditions (Friedman’s) for the three personalities, according to each component of the GEQ Core Module and Social Presence Module

		Competitive			Co-active			Collaborative				
	Component	Distant	Close	Between-groups comparison	Distant	Close	Between-groups comparison	Distant	Close	Between-groups comparison	Between conditions	
		Mdn (IQR)	Mdn (IQR)	Mann-Whitney U, *p* Value	Mdn (IQR)	Mdn (IQR)	Mann-Whitney U, *p* Value	Mdn (IQR)	Mdn (IQR)	Mann-Whitney U, *p* Value	Distant (Chi-Square, *p* Value)	Close (Chi-Square, *p* Value)
Core Module	Competence	2.50 (1.40)	2.20 (1.80)	163, 0.495	2.50 (1.05)	2.80 (1.40)	141.5, 0.195	2.20 (1.25)	2.40 (1.10)	171.5, 0.659	1.684, 0.431	0.989, 0.616
	Sensory and Imaginative Immersion	2.25 (0.95)	2.33 (1.42)	171, 0.649	2.33 (0.84)	2.50 (1.26)	155, 0.363	2.42 (1.33)	2.33 (0.83)	178.5, 0.809	1.615, 0.446	0.129, 0.938
	Flow	2.60 (1.05)	3.20 (1.10)	122.5, 0.066	2.80 (1.40)	3.00 (1.10)	142.5, 0.206	3.00 (1.20)	2.80 (1.00)	179, 0.820	5.474, 0.065	2.103, 0.349
	Tension/Annoyance	0.00 (0.00)	0.00 (0.00)	172, 0.419	0.00 (0.00)	0.00 (0.00)	182, 0.788	0.00 (0.00)	0.00 (0.00)	185, 0.882	2.000, 0.368	1.400, 0.497
	Challenge	0.90 (0.75)	0.80 (0.60)	165, 0.529	0.60 (1.25)	0.40 (1.00)	176.5, 0.764	0.60 (0.85)	0.80 (0.90)	164, 0.512	4.554, 0.103	1.276, 0.528
	Negative Affect	0.00 (0.00)	0.00 (0.00)	151.5, 0.173	0.00 (0.00)	0.00 (0.00)	153, 0.067	0.00 (0.00)	0.00 (0.00)	163, 0.242	4.261, 0.119	2.000, 0.368
	Positive Affect	3.00 (0.85)	3.00 (1.30)	154, 0.344	3.00 (1.00)	3.00 (1.00)	175.5, 0.742	3.00 (1.25)	2.83 (1.00)	159, 0.424	0.685, 0.710	0.367, 0.832
Social Presence Module	Empathy	**2.25 (1.37)**	**2.83 (1.25)**	**113.5, 0.037**	2.67 (1.25)	2.67 (0.59)	142.5, 0.203	2.75 (1.09)	3.00 (0.67)	135.5, 0.143	4.075, 0.130	3.079, 0.214
	Negative Feelings	0.50 (1.05)	0.80 (1.20)	165.5, 0.536	0.60 (1.20)	0.40 (1.10)	157.5, 0.394	0.40 (1.20)	1.00 (0.80)	139, 0.165	0.400, 0.819	2.151, 0.341
	Behavioral Involvement	**0.92 (1.04)**	**1.33 (0.84)**	125, 0.078	**1.00 (0.66)**	**1.33 (1.17)**	162.5, 0.484	**1.75 (1.42)**	**1.67 (0.59)**	185.5, 0.966	**13.835, 0.001**	**7.429, 0.024**

Bold values represent that significant differences between conditions were found

When comparing both subgroups in the same condition, we found significant differences in Empathy for the Competitive mode (U = 113.500, *p* = 0.037). Those who reported having a closer relationship (Mdn = 2.83 (1.24)) displayed higher empathy than those that reported having a distant relationship (Mdn = 2.25 (1.38)).

## Discussion

Here we compared three different game modes (Competitive, Co-active and Collaborative) to understand differences in engagement and social involvement, with the purpose of identifying the most adequate multi-player game strategy for a stroke motor rehabilitation program. However, before addressing the study with stroke survivors, we decided to study healthy elderly participants first because it is important to first assess a population on the same age range as the majority of people with stroke [30], and without significant deficits, besides those related to aging, that could act as confounding factors for the study, such as motor impairments. We acknowledge that for the selected population the main goal or motivation for playing serious games is different from who plays for rehabilitation purposes. However, we believe that the most important is the existence of motivation to engage in an activity. If this engagement is natural and voluntary, levels of motivation will be high whether for entertainment or rehabilitation. Thus, this is a first step towards understanding the impact of game modes on engagement and social interaction with stroke patients.

We found evidence that the Collaborative mode promotes significantly more Empathy and Behavioral Involvement when compared to Co-active and Competitive modes. This could mean that collaboration promotes the feeling that one’s actions depend on the co-player actions [17], leading to higher levels of attention towards others. In turn, we assume this higher awareness from the other can potentially stimulate social involvement. We hypothesize that the major reason for this result is because in a Collaborative mode a player can not win independently. In this mode, players have to help each other achieving a common goal (e.g., in this specific task, after catching one ball, they could inform which color the co-player should catch to score 1 point). Having both players contributing equally to achieve the goals enhances positive social interaction [17]. This is an important result because of recent evidence that highlights the importance of social engagement on health and well-being of older people [1]. Furthermore, social interaction in the form of multiplayer games has been described as a potentially important element to promote motor rehabilitation [31]. The Collaborative mode also potentiates more empathy, which is very important to facilitate social interaction and as well to affiliate and form social bonds [32]. Hence, multi-player serious games that require collaboration could be a relevant approach to consider for social rehabilitation.

The observed outcomes in behavioral involvement promoted by the Collaborative mode, are expected to increase engagement, as it is predictable that participants with lower skills are going to be assisted by their co-player when presenting difficulties and consequently achieve more success while the participant with higher skills is going to be more challenged and/or required to adopt an altruistic behavior [33]. Gorsic et al. [22] studied the impact of Single Player Vs Cooperative Vs Competitive game modes in motivation and exercise intensity, with unimpaired and healthy (familiar or therpaist) participants. Their description of the two used variants of Cooperative mode are actually what Mace et al. [17] describe as Collaborative (Cooperative with split field) and Co-active (Cooperative with shared field) modes, definitions that we used as guidelines. Their results identified the Collaborative mode as the less preferred and the one where participants felt less competent [22]. Similarly to their study, our results regarding the Collaborative mode also reveal this mode as the one where participants felt less competent. This indicates that serious games for rehabilitation to be played in collaboration must be carefully designed, ensuring compensation mechanisms in order to balance differences of motor and cognitive skills between participants [17].

Globally, ratings of Negative Feelings and Negative Affect were low on every mode, and in line with the high ratings of Positive Affect, which can mean the overall impact of the game modes tested was positive. The sense of Flow was generally reported as high but not significantly different among the three game modes. Also, the tasks were not considered challenging, and the sense of competence was reported as slightly positive. As levels of challenge were low, according to the Flow model [34], it would be expectable that participants would feel bored. We hypothesize that one contribution to the reported high levels of Flow was the inexistence of any kind of negative feedback through the game and constant positive feedback, as positive competence feedback is positively related to subsequent motivation [35]. The only objective informations that participants could use from the game to establish a low performance, were the balls that they could not catch, and the final score of each round with which they could infer if it was lower or higher than the previous. On the other side, constant positive feedback was being given when a ball was caught (visually through green fireworks and a “+ 1” point that appeared on the screen). Despite this positive feedback was very frequent, the sense of competence was described only as slightly positive.

Cognitive skills are important to have in consideration when using virtual rehabilitation with patients that suffered a stroke, as the capacity to understand and solve tasks have an impact on the experience [36]. Differences in the Behavioral Involvement component regarding the Collaborative mode seem to be explained by participants who had higher cognitive skills. Indeed, a study with persons that suffered a stroke, reported that patients with higher cognitive deficits rated poorly the virtual environment designed for rehabilitation [36] compared to those with lower cognitive deficits. In our opinion, the Collaborative mode is more cognitively demanding when compared to others. That is because participants need to coordinate strategies, requiring thus much more attention from the players. Additionally, since participants with higher cognitive skills may understand this better and may be required to assume a leading role, giving more instructions than receiving, it is understandable that they could feel more behavioral involved than the co-player [36].

Personality is directly linked with our social posture and how we interact with others. For that reason, we characterized our sample in three kinds of personality: average ones, those more extrovert, and those more introvert. Gorsic et al. did not find significant differences between personality scores and the game modes studied [22]. However, our results show that the most extrovert participants benefit more from the Collaborative mode compared to Competitive and Co-active modes in Behavioral Involvement. This is in line with findings reported by Novak et al. where gameplay in a Competitive and Cooperative settings can be either fun or frustrating according to different personalities [8]. Our results support that these participants felt more comfortable to interact with the co-player in a situation where they had to play as a team. Also, the more extrovert participants felt significantly more empathy with the Collaborative mode when compared to Co-active and Competitive modes. Overall, the Competitive setting was the weakest mode among the three studied to promote this kind of interaction with this specific subgroup.

Regarding participants’ previous relationship between co-players, we found that those with a distant relationship preferred to play the Collaborative mode when compared to Competitive and Co-active, which reinforces this mode as particularly suitable to promote social interaction between players. This is an important result because most of the times, in rehabilitation settings, patients do not have any previous relationship, and the use of collaborative tools can be a valid solution to foster their interaction. Moreover, results also show that the Competitive mode can be more suitable for participants that have a previously established relationship, as they feel more empathy. This result is somehow expected, as it is typically easier to deal with victory and defeat when dealing with a person that we already know. This is consistent with literature supporting that the Competitive mode is preferred to Cooperative in persons that have close relationships [5].

As limitations of this study, it would have been valuable to include open-ended questions in our assessment measures to gather additional input from the participants concerning their preferences, which mode they felt more willing to interact with the co-player, which mode they felt more motivated or which mode triggered more frustration. This kind of information could be interesting for the analysis, instead of relying just on the GEQ scores, as it would allow participants to spontaneously comment about specificities of the game and game modes, originating valuable qualitative data. Additionally, the short time duration of each experimental condition could be a limitation, as with longer times the effects could be stronger. Nonetheless, we opted for shorter durations to avoid confounds brought by fatigue or decreasing interest. Another limitation is the possible lack of generalization of results, regarding the sample. While we opted to study a sample of older adults without motor deficits to avoid confounds at this stage, we cannot ascertain that the obtained results are fully generalizable to stroke survivors. Hence, the next step is to validate these results with a sample of stroke survivors. Additionally, the specific design of the game also has potential implications for the generalization of results [36], as different games could lead to different results. Hence, caution should be taken when assuming generalization, specially when game mechanics are based on different sets of motor and/or cognitive skills and different modes of interaction among players to the ones studied here. Finally, it would have been interesting to understand how potentially unbalanced pairs in terms of skills in the games could have affected the results.

## Conclusions

This study compared the impact that three different game modes (Competitive, Co-active and Collaborative) can have in engagement and social involvement in multiplayer settings. Data showed that the Collaborative mode can have a significant positive impact on social involvement when compared to Competitive and Co-active modes. This impact seems bigger for some specific profiles, such participants without cognitive deficits and participants that are more extrovert. During collaboration, participants without cognitive deficits feel more Empathy and Flow compared to those with higher cognitive deficits. The more extrovert participants feel more empathy and are behaviorally more involved when playing the Collaborative mode. No significant differences were found regarding engagement. Further studies to extrapolate these conclusions to clinical populations are needed, as these multiplayer games are to be used for rehabilitation purposes.

## Data Availability

The descriptive measures, Friedman’s test, Mann-Whitney U Test, Kruskal-Wallis Test from which the conclusions are drawn are provided in the article. Raw data is available from the corresponding author on reasonable request.

## Abbreviations

AnTS: Analysis and Tracking System
GEQ: Game Experience Questionnaire
H MoCas: Higher Montreal Cognitive Assessments (participants with a score higher than the average)
IQR: Interquartile Range
L MoCas: Lower Montreal Cognitive Assessments (participants with a score lower than the average)
Mdn: Median
Mini-IPIP: Short version of International Personality Item Pool
MoCA: Montreal Cognitive Assessment

## References

[1] Nguyen TTH, Ishmatova D, Tapanainen T, Liukkonen TN, Katajapuu N, Makila T, et al. Impact of serious games on health and well-being of elderly: a systematic review. 2017; Available from: http://aisel.aisnet.org/hicss-50/hc/senior_use_of_health_it/5/

[2] Pollock A, Farmer SE, Brady MC, Langhorne P, Mead GE, Mehrholz J, et al. Interventions for improving upper limb function after stroke. In: The Cochrane Collaboration, editor. Cochrane database of systematic reviews. Chichester: John Wiley & Sons, Ltd; 2014. Available from: http://doi.wiley.com/10.1002/14651858.CD010820.pub2 10.1002/14651858.CD010820.pub2 PMC6469541 25387001

[3] Lohse K, Shirzad N, Verster A, Hodges N, Van der Loos HM (2013). Video games and rehabilitation: using design principles to enhance engagement in physical therapy. J Neurol Phys Ther.

[4] Northcott S, Moss B, Harrison K, Hilari K (2016). A systematic review of the impact of stroke on social support and social networks: associated factors and patterns of change. Clin Rehabil.

[5] Flores E, Tobon G, Cavallaro E, Cavallaro FI, Perry JC, Keller T (2008). Improving Patient Motivation in Game Development for Motor Deficit Rehabilitation. Proceedings of the 2008 International Conference on Advances in Computer Entertainment Technology.

[6] Simning A, Seplaki CL, Conwell Y (2016). Variation by social support in the risk for depression following a heart attack or stroke: preliminary findings from the National Health and aging trends study. Am J Geriatr Psychiatry.

[7] Janssen H, Ada L, Bernhardt J, McElduff P, Pollack M, Nilsson M (2014). Physical, cognitive and social activity levels of stroke patients undergoing rehabilitation within a mixed rehabilitation unit. Clin Rehabil.

[8] Novak D, Nagle A, Keller U, Riener R (2014). Increasing motivation in robot-aided arm rehabilitation with competitive and cooperative gameplay. J Neuroengineering Rehabil.

[9] Jarrassé N, Charalambous T, Burdet E (2012). A framework to describe, analyze and generate interactive motor behaviors. PLoS One.

[10] Sawers A, Ting LH (2014). Perspectives on human-human sensorimotor interactions for the design of rehabilitation robots. J Neuroengineering Rehabil..

[11] Baur K, Schättin A, De Bruin ED, Riener R, Duarte JE, Wolf P (2018). Trends in robot-assisted and virtual reality-assisted neuromuscular therapy: a systematic review of health-related multiplayer games. J Neuroengineering Rehabil..

[12] Chanel G, Kivikangas JM, Ravaja N (2012). Physiological compliance for social gaming analysis: cooperative versus competitive play. Interact Comput.

[13] Eastin MS (2007). The influence of competitive and cooperative group game play on state hostility. Hum Commun Res.

[14] Schmierbach M, Xu Q, Oeldorf-Hirsch A, Dardis FE (2012). Electronic friend or virtual foe: exploring the role of competitive and cooperative multiplayer video game modes in fostering enjoyment. Media Psychol.

[15] Staiano AE, Abraham AA, Calvert SL (2012). Competitive versus cooperative exergame play for African American adolescents’ executive function skills: short-term effects in a long-term training intervention. Dev Psychol.

[16] Kivikangas JM, Kätsyri J, Järvelä S, Ravaja N (2014). Gender differences in emotional responses to cooperative and competitive game play. PLoS One.

[17] Mace M, Kinany N, Rinne P, Rayner A, Bentley P, Burdet E (2017). Balancing the playing field: collaborative gaming for physical training. J Neuroengineering Rehabil..

[18] Duckworth Jonathan, Mumford Nick, Caeyenberghs Karen, Eldridge Ross, Mayson Scott, Thomas Patrick R., Shum David, Williams Gavin, Wilson Peter H. (2015). Resonance: An Interactive Tabletop Artwork for Co-located Group Rehabilitation and Play. Universal Access in Human-Computer Interaction. Access to Learning, Health and Well-Being.

[19] Goršič M, Tran MH, Novak D. Cooperative cooking: a novel virtual environment for upper limb rehabilitation. In: 2018 40th Annual International Conference of the IEEE Engineering in Medicine and Biology Society (EMBC) IEEE; 2018 p 3602–3605. 10.1109/EMBC.2018.8513005 PMC6320235 30441156

[20] Baur K, Wolf P, Klamroth-Marganska V, Bierbauer W, Scholz U, Riener R, et al. Robot-supported multiplayer rehabilitation: feasibility study of haptically linked patient-spouse training. In: 2018 IEEE/RSJ International Conference on Intelligent Robots and Systems (IROS) IEEE; 2018 p 4679–4684.

[21] Peng W, Hsieh G (2012). The influence of competition, cooperation, and player relationship in a motor performance centered computer game. Comput Hum Behav.

[22] Goršič M, Cikajlo I, Goljar N, Novak D (2017). A multisession evaluation of an adaptive competitive arm rehabilitation game. J Neuroengineering Rehabil..

[23] Llorens R, Navarro MD, Noé E, Alcañiz M (2016). Competition improves attention and motivation after stroke. Proceedings of the 11th international conference on disability, virtual reality and associated technologies.

[24] Mathews Z, i Badia SB, Verschure P. A novel brain-based approach for multi-modal multi-target tracking in a mixed reality space. In: Proceedings of 4th INTUITION international conference and workshop on virtual reality: Citeseer; 2007.

[25] IJsselsteijn W, Poels K, de Kort YA. The game experience questionnaire: development of a self-report measure to assess player experiences of digital games: TU Eindh Eindh Neth; 2008.

[26] Freitas S, Simões MR, Alves L, Santana I (2011). Montreal cognitive assessment (MoCA): normative study for the Portuguese population. J Clin Exp Neuropsychol.

[27] Simões V de J. Adaptação e validação do teste de personalidade mini-IPIP e Big Five Inventory (BFI) em adultos portugueses [Master’s Thesis]. 2016.

[28] De Kort YA, IJsselsteijn WA, Poels K. Digital games as social presence technology: development of the social presence in gaming questionnaire (SPGQ). Proc Presence. 2007;195203.

[29] Interpreting Individual IPIP Scale Scores. [cited 2019 Jan 10]. Available from: https://ipip.ori.org/InterpretingIndividualIPIPScaleScores.htm

[30] Sousa-Uva M, Dias CM. Prevalência de Acidente Vascular Cerebral na população portuguesa: dados da amostra ECOS 2013. 2014.

[31] Johnson MJ, Feng X, Johnson LM, Ramachandran B, Winters JM, Kosasih JB. Robotic systems that rehabilitate as well as motivate: three strategies for motivating impaired arm use. In: The first IEEE/RAS-EMBS international conference on biomedical robotics and biomechatronics, 2006 BioRob 2006. IEEE; 2006. p. 254–259.

[32] Shamay-Tsoory S, Lamm C. The neuroscience of empathy–from past to present and future. Elsevier; 2018. 10.1016/j.neuropsychologia.2018.04.034 29709581

[33] Nakamura J, Csikszentmihalyi M. The concept of flow. In: Flow and the foundations of positive psychology: Springer; 2014. p. 239–63.

[34] Csikszentmihalyi M. Beyond boredom and anxiety: experiencing flow in work and play: Wiley; 2000. p. 282.

[35] Csikszentmihalyi Mihaly, Abuhamdeh Sami, Nakamura Jeanne (2014). Flow. Flow and the Foundations of Positive Psychology.

[36] Mihelj M, Novak D, Milavec M, Ziherl J, Olenšek A, Munih M (2012). Virtual rehabilitation environment using principles of intrinsic motivation and game design. Presence Teleoperators Virtual Environ.

